# The clinical condition rather than cerebrospinal fluid levels may guide the optimal duration of antimicrobial therapy among neonates with bacterial meningitis: A single‐center retrospective study

**DOI:** 10.1002/pdi3.43

**Published:** 2023-11-01

**Authors:** Ya Hu, Xingyuan Li, Hong Wei, Yuanyuan Luo, Hongdong Li

**Affiliations:** ^1^ Department of Neonatology Children's Hospital of Chongqing Medical University Chongqing China; ^2^ Ministry of Education Key Laboratory of Child Development and Disorders Chongqing China; ^3^ National Clinical Research Center for Child Health and Disorders Chongqing China; ^4^ China International Science and Technology Cooperation Base of Child Development and Critical Disorders Chongqing China; ^5^ Chongqing Key Laboratory of Child Infection and Immunity Chongqing China; ^6^ Department of Pharmacy Chongqing Red Cross Hospital Chongqing China; ^7^ Department of Neurology Children's Hospital of Chongqing Medical University Chongqing China; ^8^ Department of Emergency Children's Hospital of Chongqing Medical University Chongqing China

**Keywords:** antimicrobial therapy, bacterial meningitis, cerebrospinal fluid, clinical condition, neonate

## Abstract

Neonatal bacterial meningitis is serious and accounts for high morbidity and mortality in developing countries, but the duration of antimicrobial treatment is not well established. Cerebrospinal fluid (CSF) levels are important factors for diagnosing and monitoring the occurrence and development of bacterial meningitis. In this study, we aimed to compare the clinical characteristics and outcomes of neonatal bacterial meningitis with normal CSF and abnormal CSF at discharge. We enrolled neonates with bacterial meningitis who were admitted to the neonatology department of the Children's Hospital of Chongqing Medical University between August 1, 2019 and January 1, 2021. The children's clinical data, laboratory data, and outcomes were recorded and analyzed. Fifty‐five neonates met the inclusion criteria in our study. Other than CSF protein levels, there was no significant difference in clinical symptoms, blood levels (white blood cell [WBC] count, neutrophils, platelets, C‐reactive protein, and procalcitonin), CSF WBC counts at admission and discharge, duration of antibiotics, or hospital stays between the two groups (*p* > 0.05). For the sequelae of bacterial meningitis, short‐ and long‐term complications also showed no statistically significant differences between the two groups. The duration of antimicrobial therapy in neonatal bacterial meningitis may depend on the clinical condition of the patient rather than the CSF levels. This study may improve the reasonable utilization of antibiotics in neonatal bacterial meningitis.

## Graphical abstract

There was no significant difference in clinical symptoms, blood levels, duration of antibiotics, hospital stays, and complications between neonatal bacterial meningitis with normal cerebrospinal fluid (CSF) and abnormal CSF at discharge.

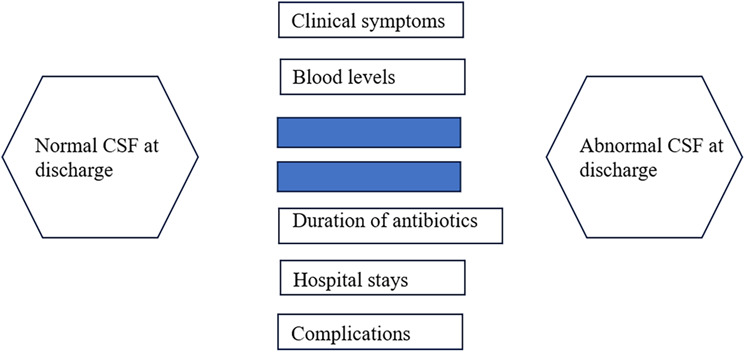

## 1 INTRODUCTION

Bacterial meningitis is a serious acute infectious disease of the central nervous system with high morbidity and mortality, especially in neonates, because of the immature blood‒brain barrier (BBB).^1^, ^2^ Neonatal bacterial meningitis can cause acute complications involving ventriculitis and subdural effusion. Moreover, long‐term neurological sequelae, including cerebral palsy, seizures, hydrocephalus, or cognitive impairment may be present in up to 50% of survivors.^3^, ^4^ Thus, early diagnosis and an adequate duration of antibiotic treatment are crucial for favorable outcomes.

Cerebrospinal fluid (CSF) culture is the gold standard for confirming neonatal bacterial meningitis.^5^ However, CSF culture often has a low yield due to the limited sensitivity of culture techniques, and we often use CSF levels (such as leukocyte count, glucose, and total protein) to diagnose bacterial meningitis. Continuous monitoring of CSF levels during antimicrobial treatment of bacterial meningitis in young infants is recommended by some specialists, which may prolong the use of antibiotics.^6^ However, there are controversies in the guidelines in the warrants regarding the indications for repeat lumbar puncture or in the explanation of the results.^7^, ^8^ In addition, prolongation of antibiotic treatment may increase the resistance of pathogenic bacteria and lead to potential diseases such as necrotizing enterocolitis, and candidiasis in neonates.^9^, ^10^ Hence, it is very important to explore the predictive value of end‐of‐therapy CSF levels in neonates.

The objective of the present study was to compare the outcomes of neonates with bacterial meningitis whose CSF levels are normal or abnormal at discharge, which could contribute to guidance for the optimal duration of antibiotics on evidence‐based data.

## 2 MATERIALS AND METHODS

### 2.1 Data collection

The study was approved by the Institutional Review Board of the Children's Hospital of Chongqing Medical University. All methods were performed in accordance with the relevant guidelines and regulations.^11^ Written informed consent to participate in this study was provided by the participants' legal guardian. Medical records were collected for neonates with bacterial meningitis by Research Electronic Data Capture. Symptoms or clinical findings were categorized as not present if they were not mentioned in the medical records. After screening, 55 neonates with bacterial meningitis were enrolled between August 1, 2019 and January 1, 2021, according to our exclusion and inclusion criteria, and all of their clinical symptoms had disappeared at discharge. Demographic characteristics, including sex, birth weight, gestational age, and admission age and maternal conditions such as vaginal delivery, meconium‐stained amniotic fluid, premature rupture of membrane, intrauterine distress, and prenatal infection, were collected. Most clinical symptoms of neonatal meningitis included the presence of fever, tachypnea, apnea, feeding difficulties, and any neurologic symptoms (seizures, hypersomnia, irritability, limb jitter, and screaming). Laboratory findings that were collected included white blood cell count (WBC), platelet (PLT), C‐reactive protein (CRP), procalcitonin (PCT), blood culture, and CSF levels (culture, WBC, protein, glucose, and chloride). The duration of antibiotic treatment, neurological‐related examination at discharge, and clinical outcomes for as long as one and a half years were also collected simultaneously.

### 2.2 Inclusion and exclusion criteria

The inclusion criteria were as follows: (1) neonates with positive CSF culture and (2) neonates with negative CSF culture but abnormal CSF levels and definite clinical manifestations. The standard of abnormal CSF levels in neonates were defined as described before: in term, babies were defined as having a CSF WBC count >32 × 10^6^/L and a protein level >1.70 g/L, while it was defined in preterm babies as a CSF WBC count >29 × 10^6^/L, a protein level >1.50 g/L with either a CSF WBC count or a protein level that met the criteria allowing for a diagnosis of bacterial meningitis.^5^ The exclusion criteria were as follows: (1) age of full‐term neonates at admission >28 days or corrected gestational age of preterm infants at admission >44 weeks, (2) hospital stay <3 days with incomplete information, (3) severe congenital malformation or hereditary metabolic diseases, (4) antibiotic therapy administered before admission, and (5) intracranial lesions such as intracranial hemorrhage or ventricular dilation.

### 2.3 Statistical analysis

Continuous variables are presented as the mean ± standard deviation or median and interquartile range. Differences in categorical variables between the groups were analyzed using the *χ* ^2^ test or Fisher's exact test. The Mann‒Whitney *U* test or *t* test was used to compare continuous variables between the groups. A *p* value <0.05 was considered to indicate statistical significance. All statistical analyses were performed using SPSS 26.0 (IBM Corp.).

## 3 RESULTS

### 3.1 Comparison of demographic characteristics and clinical symptoms of patients with normal and abnormal CSF levels at discharge

During the study period, 55 eligible neonates with bacterial meningitis were identified. There were no significant differences in gestational age, birth weight, sex, admission age, or maternal factors of patients with meningitis with and without normal CSF levels at discharge (*p* > 0.05) (Table 1). Meanwhile, the most common presenting symptoms of patients with meningitis in both groups were tachypnea (69.0% and 69.2%, respectively), hypersomnia (37.9% and 42.3%, respectively), and fever (17.2% and 23.1%, respectively). However, clinical symptoms such as fever, tachypnea, apnea, feeding difficulties, and neurologic symptoms (hypersomnia, irritability, seizures, limb jitter, and screaming) also showed no statistically significant differences between the two groups (*p* > 0.05) (Table 2).

**TABLE 1:** Demographic characteristics in neonates with bacterial meningitis.

	Abnormal‐CSF	Normal‐CSF	*p‐*value
	Group (*N* = 29)	Group (*N* = 26)	
Infantile factors			
Male gender, *n* (%)	17 (58.6)	14 (53.8)	0.721
Multiplets, *n* (%)	8 (27.6)	5 (19.2)	0.467
Preterm infant, *n* (%)	22 (75.9)	15 (57.7)	0.152
Gestational age (weeks)^a^	33.9 ± 3.9	34.6 ± 4.3	0.504
Birth weight (kilograms)^a^	2.08 ± 1.02	2.21 ± 1.06	0.431
Weight on admission (kilograms)^a^	2.0 ± 1.0	2.2 ± 0.9	0.570
Admission age (hours)^b^	3.1 (1.0, 41.0)	15.0 (1.7148.7)	0.362
Maternal factors, *n* (%)			
Vaginal delivery	7 (24.1)	9 (34.6)	0.393
Meconium stained amniotic fluid	4 (13.8)	6 (23.1)	0.490
Premature rupture of membranes	6 (20.7)	7 (26.9)	0.587
Intrauterine distress	3 (10.3)	2 (7.7)	1.000
Chorioamnionitis	3 (10.3)	3 (11.5)	1.000

Abbreviation: CSF, cerebrospinal fluid.

^a^ Data are presented as mean ± standard deviation.

^b^ Data are presented as median and interquartile range.

**TABLE 2:** Clinical symptoms in neonates with bacterial meningitis.

	Abnormal‐CSF	Normal‐CSF	*p‐*value
	Group (*N* = 29)	Group (*N* = 26)	
Fever, *n* (%)	5 (17.2)	6 (23.1)	0.589
Irritability, *n* (%)	2 (6.9)	1 (3.8)	1.000
Tachypnea, *n* (%)	20 (69.0)	18 (69.2)	0.983
Seizures, *n* (%)	1 (3.4)	0 (0.0)	1.000
Hypersomnia, *n* (%)	11 (37.9)	11 (42.3)	0.741
Apnea, *n* (%)	0 (0.0)	1 (3.8)	0.473
Limb jitter, *n* (%)	0 (0.0)	1 (3.8)	0.473
Screaming, *n* (%)	1 (3.4)	0 (0.0)	1.000
Feeding difficulties, *n* (%)	0 (0.0)	2 (7.7)	0.219

Abbreviation: CSF, cerebrospinal fluid.

### 3.2 Blood and CSF levels at admission and at discharge

In terms of laboratory parameters, the blood parameters (WBC, neutrophils, PLT, CRP, and PCT) at admission and discharge showed no significant differences between the abnormal CSF group and the normal CSF group (*p* > 0.05). The WBC count in the blood varied between 6.2 and 16.8 × 10^9^/L with a mean value of 11.5 × 10^9^/L in the abnormal CSF group at discharge, while the WBC count varied between 8.1 and 14.9 × 10^9^/L with a mean value of 11.5 × 10^9^/L in the normal CSF group. These results showed that the WBC counts had returned to normal levels at discharge, while CRP in a few neonates remained higher than 8 mg/L (6.9% and 3.8%, respectively). There was a statistically higher level of CSF protein in the abnormal CSF group than in the normal CSF group, both at the initial CSF analysis after admission (median 2.5 g/L vs. 2 g/L) and at discharge (median 2 g/L vs. 1.1 g/L) (*p* < 0.05). The CSF WBC counts showed no significant differences between the two groups at the time of admission (median 60 vs. 100 × 10^6^/L) and discharge (median 6 vs. 3 × 10^6^/L) (*p* > 0.05). In the normal CSF group, protein concentrations <1.5 g/L and WBC counts <29 × 10^6^/L were found in all neonates, indicating that the CSF levels had recovered after antimicrobial therapy.

### 3.3 Antibiotic treatment

In our study, proven meningitis (positive cultures of CSF) was obtained in only 3 (5.5%) cases (2 cases in the abnormal CSF group vs. 1 case in the normal CSF group). Positive cultures of blood were obtained in 6 (20.6%) cases in the abnormal CSF group, and the pathogens were *Escherichia coli* (2/29), *Staphylococcus epidermidis* (2/29), *Klebsiella pneumoniae* (1/29), and *Streptococcus agalactiae* (1/29). While positive cultures of blood were obtained in 5 (19.2%) cases in the normal CSF group, the pathogens were *K*. *pneumoniae* (2/26), *E*. *coli* (1/26), *Enterobacter cloacae* (1/26), and *Listeria monocytogenes* (1/26) (data not shown). Generally, the initial empirical antibiotic therapy of neonatal bacterial meningitis in our center was penicillin, combined with a third‐generation cephalosporin before bacteriological results.^12^ If multidrug‐resistant bacteria were confirmed or clinical improvement was not apparent after 48 h of antimicrobial therapy, special antibiotics such as carbapenems and glycopeptides were used. The duration of antibiotic treatment ranged from 13.5 to 33.0 days (mean 24 days) in the abnormal CSF group, while the duration of antibiotic treatment ranged from 19.0 to 36.0 days (mean 22.5 days) in the normal CSF group. This result indicated that the duration of antibiotics was at least nearly 3 weeks in the normal CSF group. However, there were no significant differences in the duration of antibiotic treatment, dual antibiotic application, or usage of carbapenems (meropenem and imipenem), and glycopeptides (vancomycin and teicoplanin) between the two groups (*p* > 0.05) (Table 3).

**TABLE 3:** Antibiotic treatment in neonates with bacterial meningitis.

	Abnormal‐CSF	Normal‐CSF	*p‐*value
	Group (*N* = 29)	Group (*N* = 26)	
Antibiotic duration (days)^a^	24.0 (13.5, 33.0)	22.5 (19.0, 36.0)	0.669
Dual antibiotics^a^	2.0 (1.0, 2.0)	2.0 (1.0, 2.0)	0.395
Usage of carbapenems^b^	25 (86.2)	25 (96.2)	0.355
Usage of glycopeptides^c^	18 (62.1)	14 (53.8)	0.537

Abbreviation: CSF, cerebrospinal fluid.

^a^ Median and interquartile range.

^b^ Carbapenems includes meropenem and imipenem.

^c^ Glycopeptides includes vancomycin and teicoplanin.

### 3.4 Short‐ and long‐term complications in infants—Sequelae after discharge from the hospital

One of these infants in each group died during the hospital stay, but they did not die of bacterial meningitis or its sequelae. One neonate in the abnormal CSF group died of gastric perforation, and another neonate in the normal CSF group died of withdrawing treatment due to severe pneumonia. Tests of infant motor performance (TIMP), amplitude integrated electroencephalogram (aEEG), video electroencephalogram (VEEG), brain‐stem auditory evoked potential (BAEP), and magnetic resonance imaging (MRI)/computed tomography (CT) at the time of discharge were routinely used to evaluate the short‐term complications of neonates. All neonates in the two groups had low TIMP scores at discharge, and more than half of the neonates in the two groups had abnormal CT/MRI results (57.6% and 56.5%, respectively). None of the neonates in either group had a recurrence of meningitis during follow‐up (data not shown). There were no significant differences in length of hospitalization, TIMP score, abnormal aEEG/VEEG, abnormal BAEP, or abnormal MRI/CT between the two groups (*p* > 0.05) (Table 4). Long‐term complications of bacterial meningitis, such as cerebellar atrophy, encephalomalacia, hydrocephalus, seizure disorder, subdural empyema, and cerebral palsy, were called neurological sequelae and showed no significant differences between the two groups (Table 4).

**TABLE 4:** Neurological related examination and clinical outcomes in neonates with bacterial meningitis at discharge.

	Abnormal‐CSF	Normal‐CSF	*p‐*value
	Group (*N* = 29)	Group (*N* = 26)	
Hospital stay (days)^a^	36.0 (21.0, 58.0)	37.0 (20.8, 60.3)	0.887
Death, *n* (%)	1 (3.4)	1 (3.8)	1.000
TIMP (*Z*)^a^	−0.8 (−1.3, −0.0)	−1.0 (−1.5, −0.6)	0.843
Abnormal aEEG/VEEG, *n* (%)	6 (20.7)	4 (15.4)	0.733
Abnormal BAEP, *n* (%)	4 (13.8)	1 (13.8)	0.355
Abnormal CT/MRI, *n* (%)^b^	19 (65.5)	15 (57.7)	0.551
Long‐term neurological sequelae during follow‐up, *n* (%)	4 (13.8)	4 (15.4)	1.000

Abbreviations: aEEG, amplitude integrated electroencephalogram; BAEP, brain‐stem auditory evoked potential; CSF, cerebrospinal fluid; CT, computed tomography; MRI, magnetic resonance imaging; TIMP, test of infant motor performance; VEEG, video electroencephalogram.

^a^ Data are presented as median and interquartile range.

^b^ Abnormal CT/MRI including hydrocephalus, encephalomalacia, and cerebral hemorrhage.

## 4 DISCUSSION

Neonatal bacterial meningitis is associated with substantial morbidity and mortality and requires appropriate use of antibiotics. The guidelines for the duration of antimicrobial therapy are based on the etiology of neonatal bacterial meningitis. Generally, a duration of 14 days was reported for group B streptococcus, *L. monocytogenes* and *Streptococcus pneumonia* meningitis, while a duration of 21 days was reported for *Pseudomonas* and gram‐negative microorganisms.^13^, ^14^ Longer antimicrobial courses are recommended for infants with meningitis with complications such as brain abscesses, ventriculitis, or brain infarctions.^14^ However, the incidence of CSF culture‐confirmed meningitis is quite low, indicating that these negative culture patients receive antimicrobial therapy empirically by physicians. Moreover, neonates with an initial positive CSF culture had persistent positive bacterial cultures on the second CSF sample, which might prolong antimicrobial therapy.^15^ In our study, we found that the adverse outcomes in neonates whose clinical symptoms disappeared at discharge but who had abnormal CSF levels were not significantly different from those in neonates with normal CSF levels at discharge. This suggests that the duration of antibiotics for bacterial meningitis depends on the clinical condition of the patient rather than the CSF levels. In fact, Zhou et al. found that if an effective course of antibiotics is sufficient for a child with bacterial meningitis, antibiotics may be discontinued, and the CSF may be regularly followed up and rechecked.^16^ We also repeated lumbar punctures after discharge for surveillance of the recurrence of bacterial meningitis once a week.

The duration of antibiotic utilization with neonatal bacterial meningitis remains controversial. The duration of antibiotics is long and frequently associated with neurological sequelae, even though the CSF is sterile and the baby has no clinical symptoms, because the neonatologist fears premature discontinuation of therapy.^17^ A multicenter retrospective study showed that longer than 3 weeks of administration of third‐generation cephalosporin had no impact on the neonate's prognosis.^12^ Mathur et al. compared the effectiveness of a 10‐day versus a 14‐day course of antibiotic therapy in neonates with meningitis and suggested that a course of antibiotic therapy as short as 10 days is effective and associated with lower mortality and adverse outcomes.^18^ In our study, we found that the duration of antibiotic treatment, even the utilization of special antibiotics such as carbapenems and glycopeptides, showed no significant difference between the two groups. Excessive use of antibiotics in neonates is one of the major risk factors for the development of antibiotic resistance and the side effects of decreased bacterial biodiversity and modulation of host metabolism.^19^, ^20^ In addition, excessive use of antibiotics prolongs hospital stays and aggravates the substantial economic burden of the patients.^21^

CSF culture is the gold standard for confirming neonatal bacterial meningitis, but the combination of deeper interpretation of CSF levels and clinical symptoms is also important for diagnosing bacterial meningitis because of the difficulty in obtaining positive cultures.^22^, ^23^, ^24^ Surveillance of CSF culture or levels relies on repeated lumbar puncture during antimicrobial therapy, which has been advocated by some experts before.^6^ However, a survey across the northwest of England revealed that only 18.2% of pediatricians routinely repeated lumbar punctures in neonatal meningitis patients.^25^ The adverse outcomes in the current study showed no significant difference between the two groups with or without normal CSF levels at discharge, indicating that the preferred option of repeating the lumbar puncture is based on the clinical condition. Following the management guidelines published by the Infectious Disease Society of North America, repeated lumbar puncture is recommended for patients who do not have apparent clinical improvement after 48 h of antimicrobial therapy.^13^, ^26^

Our study also found that only CSF protein had a significant difference between the two groups, both at the initial CSF analysis after admission and at discharge, and the results showed that CSF protein in the abnormal CSF group was higher than the normal ranges during the hospital stay (the lowest was 1.7 g/L in the current study). Protein concentrations were relatively stable in children and adolescents owing to their solid BBB. When meningitis occurs in neonates with an immature BBB, the disruption and increased permeability of the BBB lead to increased CSF protein concentrations.^27^ Tan et al. revealed that high CSF protein after 2 weeks of antimicrobial therapy was associated with poor outcome from 0 to 3 months of age, which is contrary to our results.^28^ The reason for our disputation is that the difference in the values between the groups at the time of discharge is not a finding of this study but a direct consequence of the definition of the groups.

There were some limitations to our study. First, it was a retrospective study, leading to potential bias when collecting clinical data from our medical charts. Some patients failed to receive follow‐up after discharge from the hospital. Second, all our data were obtained from a single center, leading to relatively small sample sizes, and larger sample sizes may provide more convincing results. Third, we excluded neonates who had received antimicrobial treatment prior to admission in our study, as some researchers have found that antibiotics prior to admission reduce CSF‐positive culture rates by nearly 30%.^22^, ^29^ Hence, further research is needed to confirm whether such relevance exists in our study.

In conclusion, this study demonstrated that there were no significant differences in the length of hospitalization or the prognosis of short‐ and long‐term complications in patients with or without normal CSF levels at discharge, indicating that the duration of antibiotic therapy in neonatal bacterial meningitis may depend on the clinical condition of the patient rather than the CSF levels. Further multicenter studies with large sample sizes of clinical data could be implemented to validate the results.

## AUTHOR CONTRIBUTIONS

Ya Hu was involved in conceptualization, data curation, and investigation. Yuanyuan Luo, Xingyuan Li, and Hong Wei helped in data curation, software, and formal analysis. Hongdong Li contributed to conceptualization, writing, supervision, and validation.

## CONFLICT OF INTEREST STATEMENT

The authors declare that the research was conducted in the absence of any commercial or financial relationships that could be construed as a potential conflict of interest.

## ETHICS STATEMENT

The study was approved by the Institutional Review Board of the Children's Hospital of Chongqing Medical University. All methods were performed in accordance with the relevant guidelines and regulations.^11^ Written informed consent to participate in this study was provided by the participants' legal guardian.

## Data Availability

Data sharing is not applicable to this article as no new data were created or analyzed in this study.

## References

[1] Kuti BP , Bello EO , Jegede TO , Olubosede O . Epidemiological, clinical and prognostic profile of childhood acute bacterial meningitis in a resource poor setting. J Neurosci Rural Pract. 2015;6(4):549‐557. 26752902 10.4103/0976-3147.165424 PMC4692015

[2] van de Beek D , Brouwer MC , Koedel U , Wall EC . Community‐acquired bacterial meningitis. Lancet. 2021;398(10306):1171‐1183. 34303412 10.1016/S0140-6736(21)00883-7

[3] Okike IO , Johnson AP , Henderson KL , et al. Incidence, etiology, and outcome of bacterial meningitis in infants aged <90 days in the United Kingdom and Republic of Ireland: prospective, enhanced, national population‐based surveillance. Clin Infect Dis. 2014;59(10):e150‐e157. 24997051 10.1093/cid/ciu514

[4] Thigpen MC , Whitney CG , Messonnier NE , et al. Bacterial meningitis in the United States, 1998‐2007. N Engl J Med. 2011;364(21):2016‐2025. 21612470 10.1056/NEJMoa1005384

[5] Wang H , Zhu X . Cerebrospinal fluid culture‐positive bacterial meningitis increases the risk for neurologic damage among neonates. Ann Med. 2021;53(1):2199‐2204. 34787529 10.1080/07853890.2021.2004318 PMC8604535

[6] Ouchenir L , Renaud C , Khan S , et al. The epidemiology, management, and outcomes of bacterial meningitis in infants. Pediatrics. 2017;140(1). 10.1542/peds.2017-0476 28600447

[7] Durack DT , Spanos A . End‐of‐treatment spinal tap in bacterial meningitis. Is it worthwhile. JAMA. 1982;248(1):75‐78. 7087096

[8] Ting JY , Roberts A , Khan S , et al. Predictive value of repeated cerebrospinal fluid parameters in the outcomes of bacterial meningitis in infants <90 days of age. PLoS One. 2020;15(8):e0238056. 32857801 10.1371/journal.pone.0238056 PMC7454955

[9] Cotten CM , Taylor S , Stoll B , et al. Prolonged duration of initial empirical antibiotic treatment is associated with increased rates of necrotizing enterocolitis and death for extremely low birth weight infants. Pediatrics. 2009;123(1):58‐66. 19117861 10.1542/peds.2007-3423 PMC2760222

[10] Chen J , Hu N , Xu H , et al. Molecular epidemiology, antifungal susceptibility, and virulence evaluation of Candida isolates causing invasive infection in a tertiary care teaching hospital. Front Cell Infect Microbiol. 2021;11:721439. 34604110 10.3389/fcimb.2021.721439 PMC8479822

[11] Yu X , Chen J , Tang H , et al. Identifying Prokineticin2 as a novel immunomodulatory factor in diagnosis and treatment of sepsis. Crit Care Med. 2022;50(4):674‐684. 34582411 10.1097/CCM.0000000000005335 PMC8923365

[12] Zhao Z , Hua X , Yu J , Zhang H , Li J , Li Z . Duration of empirical therapy in neonatal bacterial meningitis with third generation cephalosporin: a multicenter retrospective study. Arch Med Sci. 2019;15(6):1482‐1489. 31749877 10.5114/aoms.2018.76938 PMC6855170

[13] Ku LC , Boggess KA , Cohen‐Wolkowiez M . Bacterial meningitis in infants. Clin Perinatol. 2015;42(1):29‐45, vii‐viii. 25677995 10.1016/j.clp.2014.10.004 PMC4332563

[14] Stockmann C , Spigarelli MG , Campbell SC , et al. Considerations in the pharmacologic treatment and prevention of neonatal sepsis. Paediatr Drugs. 2014;16(1):67‐81. 24218112 10.1007/s40272-013-0057-x

[15] Di Mauro A , Cortese F , Laforgia N , et al. Neonatal bacterial meningitis: a systematic review of European available data. Minerva Pediatr. 2019;71(2):201‐208. 29160642 10.23736/S0026-4946.17.05124-6

[16] Xu JZ , Shao CC , Wang XJ , et al. circTADA2As suppress breast cancer progression and metastasis via targeting miR‐203a‐3p/SOCS3 axis. Cell Death Dis. 2019;10(3):175. 30787278 10.1038/s41419-019-1382-y PMC6382814

[17] Molyneux E , Nizami SQ , Saha S , et al. 5 versus 10 days of treatment with ceftriaxone for bacterial meningitis in children: a double‐blind randomised equivalence study. Lancet. 2011;377(9780):1837‐1845. 21620467 10.1016/S0140-6736(11)60580-1

[18] Mathur NB , Kharod P , Kumar S . Evaluation of duration of antibiotic therapy in neonatal bacterial meningitis: a randomized controlled trial. J Trop Pediatr. 2015;61(2):119‐125. 25681965 10.1093/tropej/fmv002

[19] Bailey LC , Forrest CB , Zhang P , Richards TM , Livshits A , DeRusso PA . Association of antibiotics in infancy with early childhood obesity. JAMA Pediatr. 2014;168(11):1063‐1069. 25265089 10.1001/jamapediatrics.2014.1539

[20] Ajslev TA , Andersen CS , Gamborg M , Sørensen TI , Jess T . Childhood overweight after establishment of the gut microbiota: the role of delivery mode, pre‐pregnancy weight and early administration of antibiotics. Int J Obes. 2011;35(4):522‐529. 10.1038/ijo.2011.27 21386800

[21] Thaver D , Zaidi AK . Burden of neonatal infections in developing countries: a review of evidence from community‐based studies. Pediatr Infect Dis J. 2009;28(1 suppl):S3‐S9. 19106760 10.1097/INF.0b013e3181958755

[22] Kanegaye JT , Soliemanzadeh P , Bradley JS . Lumbar puncture in pediatric bacterial meningitis: defining the time interval for recovery of cerebrospinal fluid pathogens after parenteral antibiotic pretreatment. Pediatrics. 2001;108(5):1169‐1174. 11694698

[23] Oostenbrink R , Moons KG , Derksen‐Lubsen AG , Grobbee DE , Moll HA . A diagnostic decision rule for management of children with meningeal signs. Eur J Epidemiol. 2004;19(2):109‐116. 15074565 10.1023/b:ejep.0000017828.13995.76

[24] Mintegi S , García S , Martín MJ , et al. Clinical prediction rule for distinguishing bacterial from aseptic meningitis. Pediatrics. 2020;146(3). 10.1542/peds.2020-1126 32843440

[25] Agarwal R , Emmerson AJ . Should repeat lumbar punctures be routinely done in neonates with bacterial meningitis? Results of a survey into clinical practice. Arch Dis Child. 2001;84(5):451‐452. 10.1136/adc.84.5.450d PMC1718764 11360821

[26] Tunkel AR , Hartman BJ , Kaplan SL , et al. Practice guidelines for the management of bacterial meningitis. Clin Infect Dis. 2004;39(9):1267‐1284. 15494903 10.1086/425368

[27] Huang H , Tan J , Gong X , et al. Comparing single vs. combined cerebrospinal fluid parameters for diagnosing full‐term neonatal bacterial meningitis. Front Neurol. 2019;10:12. 30728800 10.3389/fneur.2019.00012 PMC6351467

[28] Tan J , Kan J , Qiu G , et al. Clinical prognosis in neonatal bacterial meningitis: the role of cerebrospinal fluid protein. PLoS One. 2015;10(10):e0141620. 26509880 10.1371/journal.pone.0141620 PMC4625018

[29] Dalton HP , Allison MJ . Modification of laboratory results by partial treatment of bacterial meningitis. Am J Clin Pathol. 1968;49(3):410‐413. 4384575 10.1093/ajcp/49.3.410

